# Scientist’s Opinion on Climate Change and Hard Ticks (Ixodidae)

**DOI:** 10.3390/pathogens15020206

**Published:** 2026-02-12

**Authors:** Agustín Estrada-Peña, José de la Fuente

**Affiliations:** 1 Department of Animal Pathology, Universidad de Zaragoza, 50013 Zaragoza, Spain; 2 CSAI Foundation, Ministry of Human Health, 29014 Madrid, Spain; 3 SaBio (Health and Biotechnology), Instituto de Investigación en Recursos Cinegéticos IRec-csic-UclM-JccM, 13005 Ciudad Real, Spain; jose_delafuente@yahoo.com; 4 Department of Veterinary Pathobiology, Center for Veterinary Health Sciences, Oklahoma State University, Stillwater, OK 74078, USA

**Keywords:** climate, Ixodidae, model, tick, tick-borne pathogens

## Abstract

Tick-borne diseases account for a substantial proportion of the global incidence of infectious diseases, and their recent expansion has been increasingly associated with climate change. Nevertheless, previous studies have produced heterogeneous and often inconclusive results, largely due to differences in spatial scale, variable selection, and limited integration of climatic, ecological, and host-related drivers. Here, we assess the modeled impact of climate trends on the global distribution patterns of ticks parasitizing humans and livestock, rather than changes in tick abundance or pathogen transmission. This study is not an evaluation of human or animal contact rates with ticks. Using the largest curated compilation of georeferenced tick records available to date (213,513 records from 138 Ixodidae species), we adopt a global, climate-centered perspective based on the Holdridge life zones framework. The study characterized current climatic niches of tick genera and projected changes in suitability under future climate scenarios for 2040, 2060, 2080, and 2100. Our results reveal a strong association between tick occurrence patterns and large-scale gradients of temperature and atmospheric water balance, while precipitation plays a comparatively minor role. Projections indicate increasing climatic suitability for human-biting ticks at higher northern latitudes, concurrent with declining suitability across parts of central and southern Africa. By integrating modeled suitability with human population projections and livestock distributions, we estimated future changes in exposure risk. Although local processes such as tick abundance and pathogen prevalence are beyond the scope of this study, our findings provide a coherent global synthesis of how climate change may reshape tick distributions and associated risks.

## 1. Introduction

Globally, vector-borne diseases, including those transmitted by ticks, account for over 17% of all infectious diseases, causing more than 700,000 deaths annually (https://wellcome.org/insights/articles/how-climate-change-affects-vector-borne-diseases, accessed on 13 January 2026). The increase in cases is classically linked to factors such as climate change, which is associated with the expansion of the geographic range where ticks can survive and thrive. However, to date, only a few species of ticks have been explored in field studies to confirm the role of the climate trends in the changes of the range of ticks [1,2,3,4].

Studies carried out in the last two or three decades using different simulation approaches produced different overviews of the impact of a set of climate variables on the geographic range of ticks. The conclusions that can be obtained from these reports are blurred by both the scale of the studies (e.g., local or country level) and the variables chosen as the best descriptors for a given system. In any case, the general picture shows a warming world in which ticks of importance for human health are spreading north, at a variable speed according to regions, and are active during a period that was historically too cold for tick activity [5], thus extending the period of activity and increasing contact with humans [6]. At least in the USA and for the tick *Ixodes scapularis*, a link with climate remains uncertain [7] because expansion could be driven also by the abundance or the spread of vertebrates used as hosts. However, this observation may be misinterpreted. It is known that climate change is driving the changing range of many vertebrate species [8]. Thus, the direct effects of climate trends on ticks are only part of the actions that can impact the observed changes in tick distribution; the indirect actions of climate on hosts would be a second point to consider. Studies also support the view that models are still very imperfect for projecting the consequences of changes in the driving variables, e.g., [9]. This view is consistent with the lack of records of climate preferences for the three stages of a tick’s life cycle, disregarding the effects of hosts on ticks. However, it has been observed that for generalist ticks, the spatial projection of the climate niche of the species is adequate to determine the patterns of occurrence (presence—absence) [10,11,12,13], while the abundance is far from being adequately modeled if key hosts are not included in the modeling framework [14,15,16,17]. These interactions between vertebrates and ticks may be of a local or regional nature and difficult to translate to larger scales. The importance of the landscape fragmentation in the abundance of questing ticks has also been addressed [18,19].

There is accumulating proof of the direct influence of climate change on the geographic range of ticks. Laboratory tests have been completed with gradients of relative humidity or saturation deficit, using conditioned chambers to test the importance of water content and temperature on tick survival and development [20,21,22]. Studies carried out under controlled conditions in the laboratory demonstrated as part of the basics of tick biology that the speed of development of the molting and ovipositing phases is temperature-dependent. For most ticks, provided that humidity is kept under a relatively high limit (e.g., higher than 60%, and for some species, well above 80%), the temperature has an accelerating effect until a threshold is reached, when temperature is injurious for ticks and detrimental to the survival of the population. The results are coherent, but some studies reported the importance of the rain on the questing ticks [23]. Rainfall is generally associated with tick expansion and activity due to moisture and vegetation growth. Since no laboratory tests have been carried out with surrogates of rain, and all the reports have been obtained from field collections, we prefer to consider that temperature and relative humidity (or other measures of water in the air) drive the tick’s life cycle [24]. The warming of the Earth is unequivocal and unprecedented. Over the past half-century, climate warming has coincided with the spread and population increase of wildlife that may feed high loads of ticks, and there is strong evidence that, at least in the USA, wild ungulates have played a crucial role in facilitating the spread and proliferation of some ticks over the last century [7]. However, due to a lack of spatially and temporally consistent data, climate, landscape, and host variables are rarely included together in the same models at wide spatial scales [10,25,26]. Models and regional surveys demonstrated that the vertebrates are central in considerations regarding tick-borne pathogens [27], and modeling exercises point to the tight relationships of occurrence patterns of both ticks and vertebrates, delineating epidemiological regions [28] that seem to be driven by climate at some point [12].

It is known that human habits, the modifications of land cover, and the changing land use also have a deep impact on the tick’s habitat suitability. Some of these changes are mediated by human actions, such as the reforestation, the conversion of forest masses into grazing areas, etc. However, some of them are partly produced by the trends in climate change, such as desertification, the growth of rural areas, and vegetation changes [29,30,31].

Laboratory and small-scale field studies have offered insights into how temperature and humidity affect the survival and reproduction of ticks but predicting their broad-scale distribution and abundance remains a challenge. Studies have been conducted to model either abundance or occurrence of ticks at state, regional, and global scales using climate and/or landscape variables [32,33,34]. Results are nevertheless inconclusive regarding the projected burden of climate change on ticks on a world scale. Across these models, the functional relationships between seasonal or annual measures of heat, cold, precipitation, or humidity and tick presence or abundance at different scales of resolution have been inconsistent [35,36,37,38,39,40,41,42,43]. Previous efforts to focus on the problem of proposing a framework for analysis and discussion are outdated, e.g., [44].

This study examines the modeled impact of climate trends on the spatial ranges of tick species parasitizing humans and/or livestock. The primary aim is to characterize and summarize the distributions of 138 tick species under current climate conditions and under projected climates for the years 2040, 2060, 2080, and 2100. Instead of addressing a simplified scenario of potential changes in a single country or region, we adopt a global perspective based on the largest curated collection of georeferenced tick records, jointly with an integrative view of climate as the principal driver of tick distribution. We did not evaluate potential shifts in the distributions of hosts because of the logistical constraints involved in modeling tens of millions of georeferenced host records. Consequently, this study does not assess the possible redistribution of tick-borne pathogens, which largely depend on the ranges of vertebrate reservoirs and amplifying hosts. Local processes, such as changes in tick abundance or pathogen prevalence, are also beyond the scope of this work. The study is complemented with scenarios on human population distribution under future climate scenarios and an explicit evaluation of the impact on either humans or livestock from the altered patterns of tick presence.

The novelty of the present study lies in both its conceptual framework and its unprecedented taxonomic and geographic scope. Instead of relying on conventional species distribution models (SDMs), which typically estimate correlative relationships between occurrences and selected climatic predictors, we adopt the Holdridge Life Zone (HLZ) framework as an integrative representation of climate. By synthesizing temperature, precipitation, and potential evapotranspiration into ecologically meaningful units, the HLZ system is particularly suited to assessing long-term, global-scale climate-driven shifts in tick distributions.

This approach conceptually differs from traditional SDMs by emphasizing changes in climatic envelopes and life-supporting conditions, rather than species-specific response curves. Combined with the largest curated compilation of georeferenced records available for 138 tick species of medical and veterinary relevance worldwide, our analysis extends beyond previous global or continental studies that focused on a limited number of taxa or regions. Together, the HLZ framework and the scale of the dataset provide a novel and coherent perspective on how ongoing and future climate change may reshape the global distribution of ticks and their potential impacts on humans and livestock.

## 2. Materials and Methods

### 2.1. Sources of Records

The largest available global collection of tick records was assembled by compiling data from multiple sources. Only records from the family Ixodidae (hard ticks) were included because of ongoing taxonomic issues within the family Argasidae, where many species lack reliable generic assignments, and because the predominantly endophilous behavior of Argasid ticks renders the mapping of their distributions along climatic gradients inappropriate [45].

Multiple sources were consulted to compile a robust dataset of tick occurrences worldwide. Tick records were drawn from two previous compilations [11,46], species-specific datasets for Europe [47,48,49,50], and VectorMap (formerly available at https://vetormap.si.edu, accessed on 16 December 2023, currently unavailable for download). Records available as of December 2023 were incorporated from VectorMap. African tick records were updated using the collection assembled by Pierre-Claude Morel and deposited at CIRAD in Montpellier (courtesy of Laurence Vial and Frederick Stachurski). Records for the Neotropical region were updated with published data [51] with additional references provided by Alberto A. Guglielmone and Santiago Nava (INTA, Rafaela, Argentina). Data for the United Kingdom were updated using the National Biodiversity Network Trust (https://docs.nbnatlas.org/guidance-for-using-data/, accessed on 13 December 2025). Nearctic-region records were updated using material from the U.S. National Tick Collection, as available in GBIF https://www.gbif.org/, accessed on 13 December 2025, and data from the NEON project (National Ecological Observatory Network Biorepository, 2025, available at https://www.neonscience.org/data-collection/ticks, accessed on 13 December 2025). Available reports on ticks in Canada and the United States were also used [34,52], as well as a review of georeferenced records of ticks in China [53,54]. Credits and DOIs of downloads from GBIF are included in the Data Availability Statement of this paper.

The complete dataset consisted of heterogeneous formats, which were harmonized into a unified structure. For each record, we retained the species epithet, locality, country, and geographic coordinates, discarding entries with coordinate precision below three decimal places. Additional metadata, such as hosts, coordinate accuracy, and collection date, were retained when available, though not all components were used in subsequent analyses. Quality control procedures implemented in R [55] included: (a) removal of coordinates located offshore and (b) removal of records inconsistent with the known country-level distribution of the species, based on Guglielmone et al. [56]. We initially compiled distributional data on 255 species of ticks affecting humans and 212 species of ticks affecting livestock, consisting of more than 350,000 records; notably, several species are included in both groups. After cleaning of repeated records, excluding suspicious entries, and keeping only the species with more than 200 records, the final dataset comprised 213,513 records, including 198,318 georeferenced records from 79 species reported on humans and 161,764 from 59 species parasitizing livestock. Efforts were made throughout the complete protocol to have a suitable number of tick records for each species, to avoid issues of under-representation and poor modeling. As a golden rule, we did not include any species of ticks with less than 200 accurate, georeferenced, non-duplicated records. Supplementary Materials S1 includes the lists of tick species reported on humans or livestock and included in this study. Supplementary Materials S2 is a geopackage, including all the information regarding climate, ticks, and coordinates, as compiled in this study. The geopackage can be explored with open access software, such as R or qGIS (v. 3.40).

### 2.2. Human Density Data, Livestock Abundance, and Biogeographical Data

We used human population data from the SEDAC Gridded Population of the World [57], selecting grids for the baseline climate (year 2010) and future estimates for the years 2040, 2060, 2080, and 2100. Livestock density data were obtained from FAO’s Gridded Livestock of the World (https://www.fao.org/land-water/land/land-governance/land-resources-planning-toolbox/category/details/fr/c/1236449/, accessed on 12 July 2025). Human and livestock density values were resampled to the hexagonal grid.

**Figure 1:**
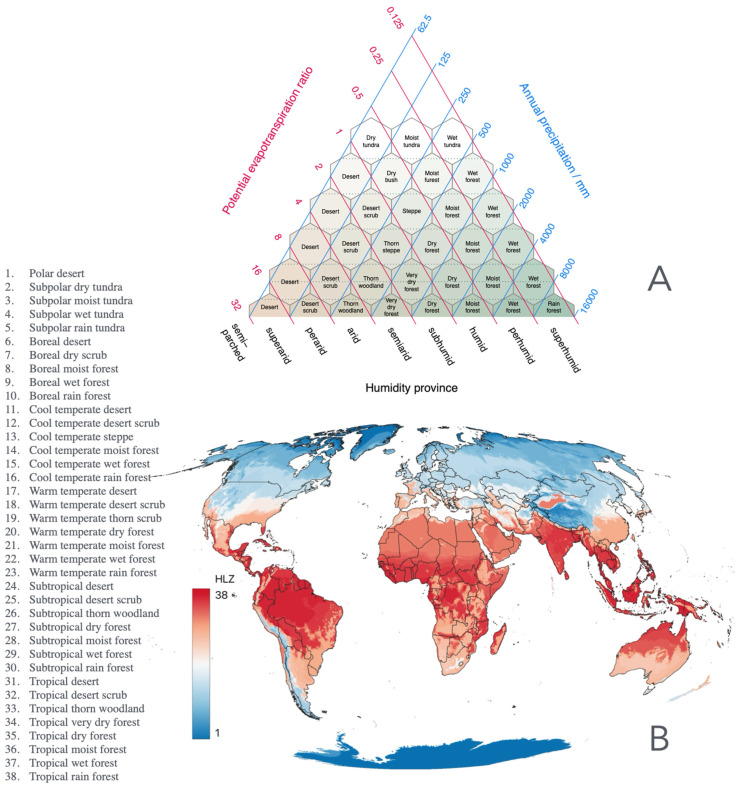
Holdridge life zones (HLZs). (**A**). The representation of the Holdridge’s life zones (HLZs) concept is in the triangle that relates the temperature, the precipitation, and the evaporation. (**B**). The spatial arrangement of the Holdridge zones, following standard values of temperature, precipitation, and evaporation.

We examined the basic relationships between ticks, climate features, and broad-scale biogeographical classifications, using the Holdridge life zones (HLZs) as a general descriptor of global biological regions (Figure 1A,B). Our objective was to identify potential indicator species, i.e., tick species consistently associated with specific climatic categories, that may serve as early markers of shifts in tick distributions. HLZ was used as the main descriptor of ecosystems and as the basis for modeling (see below). HLZ constitutes a global bioclimatic classification system defined by average annual biotemperature, meaning annual precipitation, and the potential evapotranspiration ratio. Biotemperature is an adjusted annual mean that accounts for plant metabolic limits (0–30 °C). These variables define discrete life zones that correspond to distinct ecological assemblages. To note, HLZ is a summary of the description of biomes as currently known to be associated with climate patterns and ranges of main variables, as explained. They represent macroecological reorganization rather than predictive changes in local population dynamics. We used spatial layers as provided [58] for the year 2020 (the baseline) and for the years 2040, 2060, 2080, and 2100. The scenarios of future HLZ conditions and extent are coherent with other future climate scenarios and were validated in the original publication [58].

Indicator species were identified following previous research [59]. The Indicator Value Index (IndVal) is defined as: IndVal{ij} = A{ij} × B_{ij} × 100, where A is specificity and B is fidelity. Significance was assessed using 999 permutations. Specificity is the proportion of records of species *i* that were collected in the HLZ *j*; it measures the “exclusivity” of the species for the site. Fidelity is the proportion of sites in the HLZ *j* in which the species *i* appears. Because IndVal is sensitive to the number of records and collection pressure varies by species, we restricted the analysis to species with >200 records. Calculations used the IndicSpec R package version 4.5.1 [59].

Then, we further examined the relationships of the ticks in the reduced climate niche. Climate data as used to build the HLZ were obtained for each record, using the provided coordinates. This allowed us to assemble a large dataset of climate conditions under which each species has been collected. We carried out several analyses on these premises, namely (a) a MANOVA of the associations of ticks with climate, to support the hypothesis that groups of tick genera are within bounds of climate regions; the confirmation of the contrary hypothesis would make further analysis unreliable, (b) a redundancy analysis to check if there are statistically significant associations between the HLZ and each tick genus, and how they are related in the reduced space, and (c) a principal components analysis, to relate among them groups of ticks that share a common climate niche. Supplementary Materials S2 includes a script in R with all the steps to calculate the analyses mentioned above.

### 2.3. Modeling

We approached the probability of occurrence of ticks at defined time periods according to the changes in the HLZ, calculated from the baseline scenario and with data provided for the years 2040, 2060, 2080, and 2100 as reported [58]. The aim of modeling is not the comparison of several algorithms or the association of climate variables with probabilities of occurrence, because climate data, projections of population, and other necessary data are not available at the same time periods or similar resolutions. Furthermore, the individual modeling of hundreds of species, at high resolution, for the entire Earth’s surface, is a logistical challenge. Given the explanatory nature of this study, aimed at settling a broad view of the impact of climate trends on ticks’ distribution, we addressed the projections of tick occurrence (very well represented in our dataset) against the ecological information contained in the HLZ, summarizing data for tick genera.

We applied a hybrid reconstruction method of tick occurrence, based on the projections of future changes in the climate regions of HLZ, which is based on the three basic climate variables mentioned above. We first derived an empirical genus–HLZ association from the present. For each genus *i*, we overlaid the current suitability map p_i_^curr^(x) with current HLZ classes and computed:

m_i_,𝓏 = mean_x_∈𝓏 (p_i_^curr^(x))

interpreted as the expected probability of occurrence of the tick genus *i* in zone *z* under the current climate. Future HLZ rasters (2021–2040, 2041–2060, 2061–2080, and 2081–2100) were then used as templates. Each cell assigned to HLZ z’ was filled with the corresponding value m_i_,𝓏. Thus, future suitability reflects the transfer of present-day niche–zone associations to the future range of the niche, assuming that species track their niches [60].

Using these reconstructed suitability maps, we computed expected richness (sum of genus-level suitability values), probability of occurrence of more than one genus, average suitability per HLZ, and temporal changes relative to the baseline. This approach is rapid, repeatable, and biologically interpretable, assuming temporal stability in genera–HLZ relationships. Once current and future ranges were modeled, the trend of the climate suitability for ticks, either for humans or livestock, was calculated to evaluate the areas that most changed; the range in which the suitability increased by more than 20% was separately outlined. Supplementary Materials S3 includes a script in R allowing the reproduction of the analysis detailed above.

### 2.4. Estimation of Spatial Trends and Impact

Spatial analyses were performed using 21,130 hexagonal tiles (10-km radius) covering the global terrestrial surface. Hexagons were clipped along coastlines to conform to land boundaries. These spatial units were used to quantify tick record density, species richness, human and livestock population counts, and climate summaries, all of which were required to construct indices of tick pressure on hosts (see below). Point-level tick occurrence records were assigned to their corresponding hexagons. Standard map-algebra operations were then applied to derive, for each hexagon, total human and livestock population sizes, mean values of climate variables, total numbers of tick records (calculated separately for human- and livestock-associated ticks), and the expected impact of ticks on host populations.

Each hexagon was populated with: (a) tick record density, calculated as the total number of tick records per hexagon and weighted by area; (b) species richness, defined as the number of distinct tick species recorded within each hexagon, also weighted by area; and (c) human and livestock population counts. Species richness represents the diversity of ticks biting humans or livestock and serves as a proxy for the diversity of circulating tick-borne pathogens. In addition, each hexagon contained averaged climate values, population sizes for humans and for livestock (considered separately for cattle, sheep, and goats), and the dominant HLZ classification.

For humans, tick impact was estimated using the modeled probability of occurrence of human-biting tick species combined with a categorical classification of biting frequency [60,61]. This frequency scale, ranging from “very rare” to “very frequent,” was converted into a numerical scale from 0.8 to 2.0 in increments of 0.3. Raw counts of human-biting tick records were not used due to known sampling biases. Instead, we calculated impact as modeled climate suitability (ranging from 0 to 1) multiplied by human population density and weighted by hexagon area. The same approach was applied to future climate scenarios, as projections of human population density are available for multiple time slices [57]. Modeled impacts of ticks under current and future climate conditions were calculated at the hexagon level and subsequently aggregated to the country level. For each country, we quantified gains and losses in suitable area (expressed as the total area of hexagons) and estimated overall impact by accounting for the relevance of each tick species to humans and the population density within each hexagon. This procedure was repeated for each time step using updated suitability projections and human population estimates.

For livestock, we followed the same conceptual framework; however, a classification of biting frequency was unavailable. Consequently, analyses were conducted collectively for ruminants and restricted to current conditions only. Projections under future climate scenarios were not performed for livestock due to the lack of reliable global-scale estimates of future livestock distributions. Although livestock density could, in principle, be extrapolated from climate variables, livestock distributions are also strongly influenced by socioeconomic, demographic, land-use, and land-cover factors, many of which cannot be reliably quantified at a global scale in this study. Therefore, results for ticks parasitizing livestock are presented as distributional trends rather than population-weighted impact estimates.

## 3. Results

### General Results

**Figure 2:**
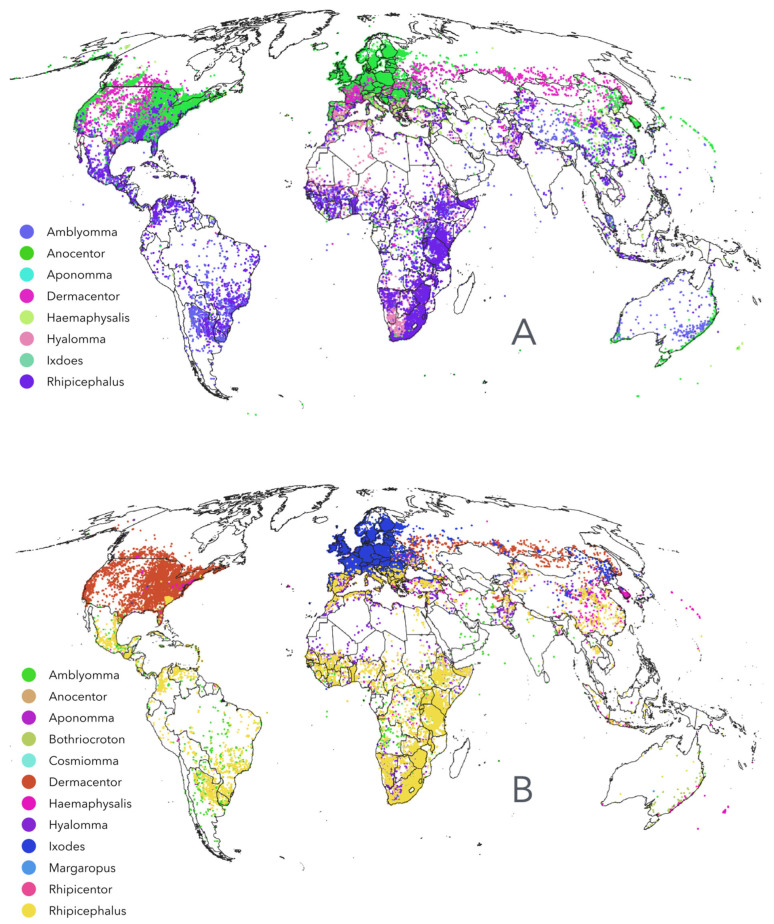
Records of ticks Ixodidae. (**A**). The spatial range of the georeferenced records of ticks compiled for this study reported them as parasites of humans. (**B**). The spatial range of the georeferenced records of ticks compiled for this study is reported as parasites of livestock, namely cattle, sheep, or goats.

A total of 213,513 records of ticks belonging to the family Ixodidae were compiled, of which 198,318 were reported from humans and 161,764 from livestock (Figure 2A,B). These maps are not intended to delimit the geographic ranges of tick genera but rather to illustrate the broad spatial coverage of the compiled records. Importantly, humans do not have specific tick parasites; all human infestations are caused by generalist species. Both humans and livestock share tick species with wildlife. Most well-surveyed regions are in the Nearctic and western Palearctic, likely reflecting the high level of awareness and surveillance in these areas. Additional well-sampled regions include livestock-producing areas of Mexico, northern Argentina, southern Brazil, and large portions of Africa, particularly around the Rift Valley. In contrast, countries such as Russia, China, and India lack substantial numbers of georeferenced tick records. The conspicuous absence of records across central and northern Russia is particularly notable.

**Figure 3:**
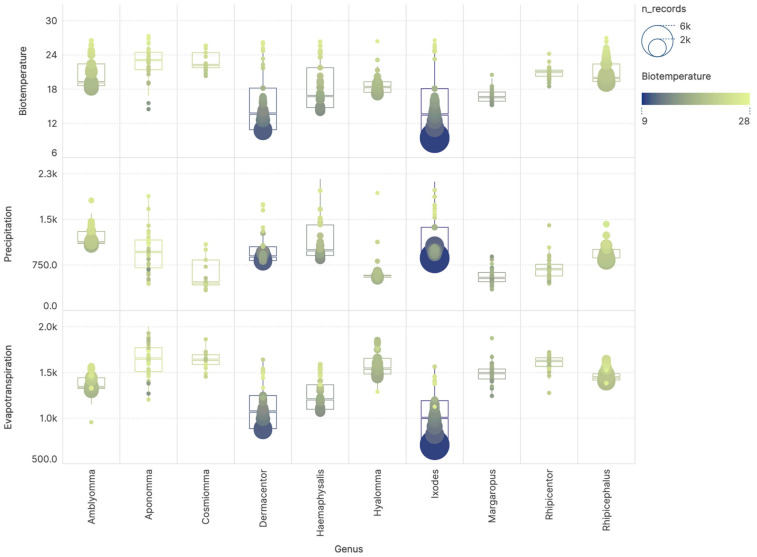
The values of biotemperature, precipitation, and evapotranspiration for the compiled records of ticks in 10 genera. Plots include dots for individual records, as well as box and whiskers plots. A pattern of color is included for the plot according to the values of biotemperature to better interpret the charts. The number of georeferenced records included in each interval of climate values is indicated by its size.

The associations between tick records and the major climatic variables used to generate the HLZ framework were examined. The results indicated that tick genera occupy distinct portions of the climatic niche (Figure 3). A MANOVA analysis revealed highly significant differences among genera with respect to biotemperature, precipitation, and evapotranspiration values associated with their records (*p* = 2.2 × 10^−16^; DF = 9; F = 20.617; Pillai’s Trace = 0.9114). Each genus shows a characteristic combination of the three variables. For some genera (*Aponomma*, *Cosmiomma*, and *Rhipicentor*), the number of available records is likely insufficient to fully capture their climatic niche, and they were removed from further calculations. Overlap among genera in certain parts of the niche likely explains their spatial co-occurrence. *Ixodes* and *Dermacentor* show preferences for the lowest temperatures, the lowest evaporation values, and a broad range of precipitation. *Haemaphysalis* occupies a slightly warmer niche with higher evaporation values. The genera *Amblyomma*, *Hyalomma*, and *Rhipicephalus* occur in the warmest and driest environments. Collectively, these findings suggest that rainfall exerts relatively weak control on the global distribution of ticks, whereas accumulated temperature above a threshold and water balance are the primary drivers of their spatial patterns.

**Figure 4:**
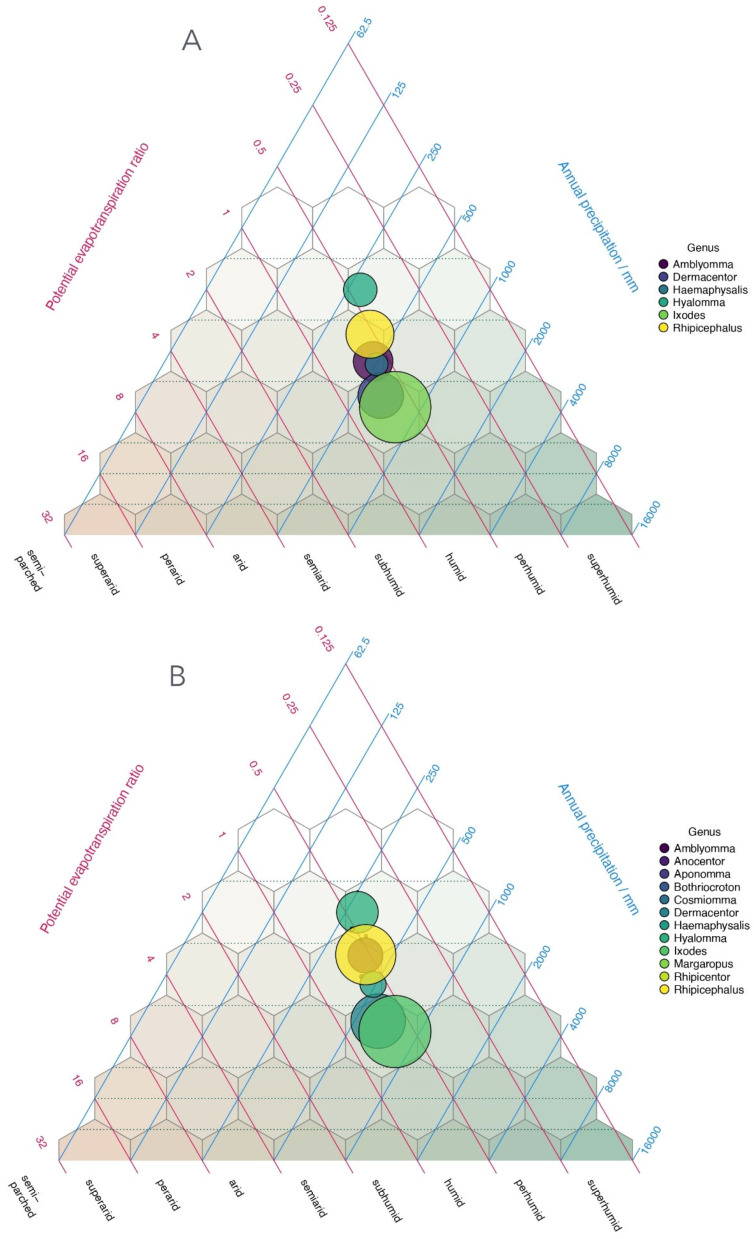

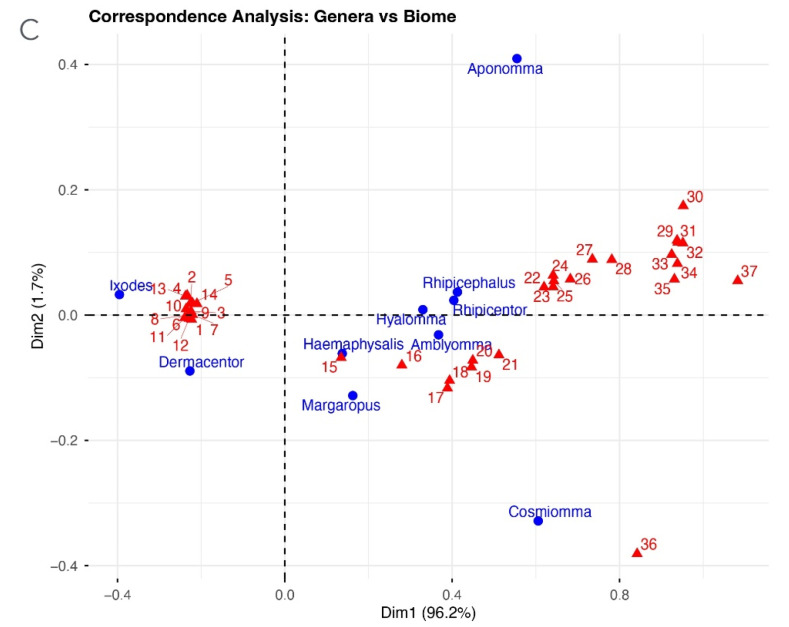
Plot of tick records. The plot of the records of ticks, either reported as parasites of humans (**A**) or livestock (**B**), on the HLZ zones built on bioclimatic variables. All the reports of each species were used to calculate the circle representing each genus in the HLZ. The size is related to the number of records of ticks. An RDA analysis (**C**) demonstrates the linking of the records of the tick genera around well-defined classes of climate (tick genera associated by distance and position with the number of the habitats in HLZ). Points in red are the numbers of the Holdridge life zones. Names in blue, are the genera of ticks.

The tick–climate relationships were translated into HLZ space by plotting the number of records for each genus within the triangular representation of biozones derived from climate values at each occurrence point (Figure 4). The distribution of tick genera associated with humans or livestock shows a clear alignment along a narrow portion of the potential evaporation ratio, ranging from <0.5 to ~1. However, ticks occur under a wide spectrum of rainfall regimes, from <250 to >1000 mm per year. More than half of all tick occurrences lie within a “humid” biozone, which spans several temperature categories, including cold temperate, temperate, subtropical, and tropical (Figure 4A,B). These patterns reflect genera-level tendencies rather than species-specific ecological requirements. An RDA conducted using tick occurrences and HLZ classes demonstrates a strong association between tick genera and specific biozones (Figure 4C), supporting the affinity of *Ixodes* and *Dermacentor* for the coldest regions and the preference of *Hyalomma* and *Rhipicephalus* for warm, dry biozones. Thus, from a global perspective and using 213,513 records of 138 tick species, there is a confirmed link between the occurrence patterns and the large climate gradients. Principal components analysis can separate the species of ticks into coherent groups of climate affinities under the premises of temperature and water in the air. The relationships with the precipitation patterns are not apparent.

However, analyses could not find good relationships between the occurrence of species as indicators of the HLZ. Table 1 shows that well-represented species can be unambiguously placed as the species that define an HLZ. For example, three Nearctic species, namely *Amblyomma americanum*, *Dermacentor variabilis*, and *Ixodes scapularis*, are good indicators of the HLZ #21. Other species define the main HLZ in Australia for ticks (HLZ #37). However, habitats in the Afrotropics and Neotropics do not have indicator species, either alone or in combination, as pairs or triads of species. We conclude that the method is ecologically sound, but the lack of a random design for tick collections, together with a lack of homogeneity in the number of available records per species, is behind the relative lack of explanatory power for some regions.

**Figure 5:**
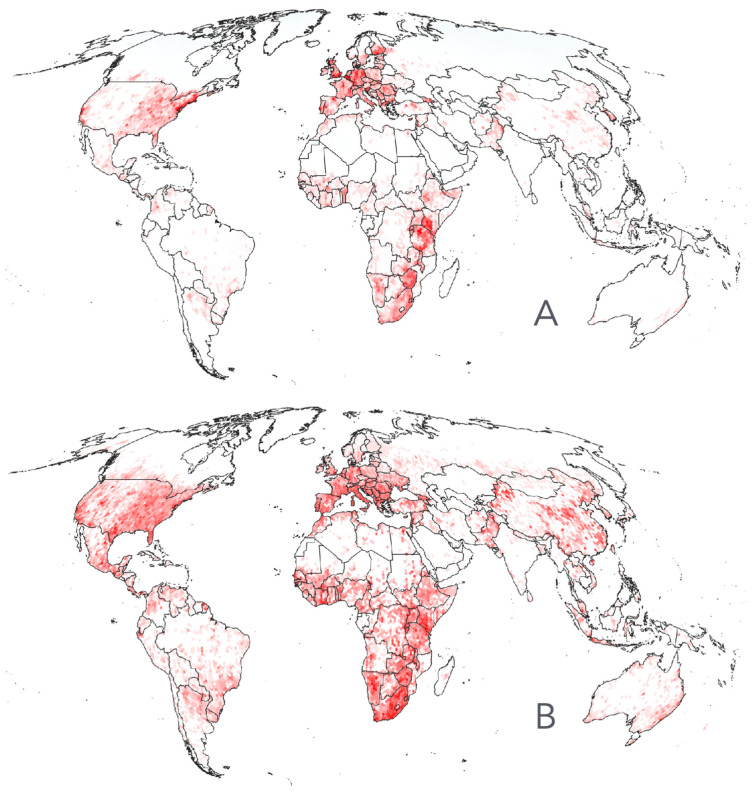
Tick density and richness. (**A**). The density of records of ticks affecting either humans or livestock. (**B**). The richness of species of ticks affecting either humans or livestock. Both tick density and richness are displayed in tones of red (the highest the darkest).

We calculated the density of reports of ticks affecting humans or livestock (Figure 5A,B), defined as the number of records per hexagon weighted by hexagon size. High density values indicate both greater tick occurrence and more intensive georeferenced surveying. The highest densities are observed across most of Europe (excluding the far north), sub-Saharan Africa, and much of the Nearctic, excluding Canada. High densities also occur in parts of the Neotropics, China, and Australia. Large areas of Russia show extremely low record density, indicating a lack of published, georeferenced data. Similarly, India has very few georeferenced tick records, resulting in an incomplete representation of its fauna. Species richness displays a spatial pattern broadly similar to record density (Figure 5B). The highest richness values occur in well-surveyed regions of Europe, North America, and parts of Africa. Interestingly, China exhibits low record density but high species richness, suggesting limited georeferenced reporting but high taxonomic diversity in the available surveys. The recorded distribution of ticks overlaps with approximately 46% of the global human population distribution, considering both human population density and tick occurrence. More than 10^9^ people live in areas of medium-to-high suitability for ticks.

**Figure 6:**
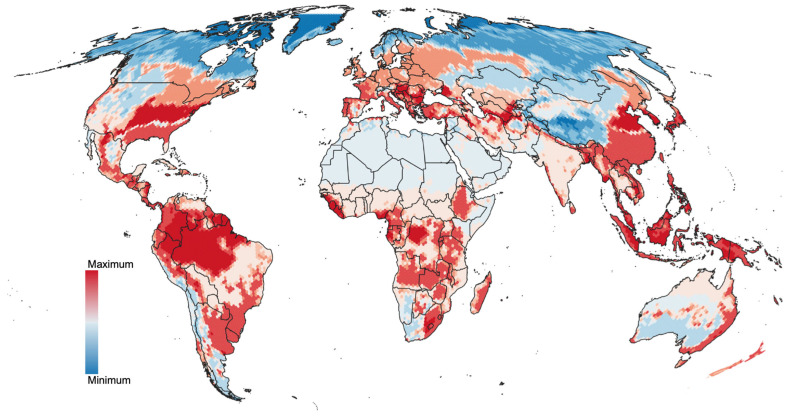
Climate suitability under current conditions. Data summarized between 1990 and 2020 for the species of ticks that affect humans.

**Figure 7:**
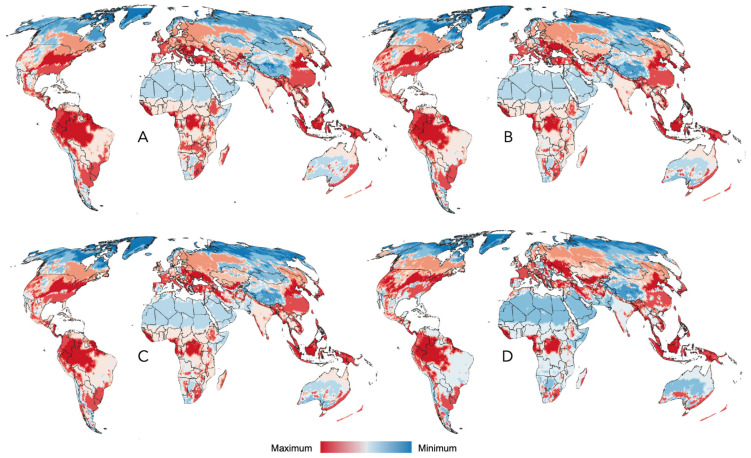
Modeled changes in climate suitability for tick species that bite humans. The modeled changes in climate suitability for the species of ticks reported to bite humans in the time periods 2040 (**A**), 2060 (**B**), 2080 (**C**), and 2100 (**D**). There is a sustained trend of increasing suitability in northern latitudes and Australia, with a lack of suitable zones in Neotropical and Afrotropical regions.

The suitability of the compiled records, the spatial randomness of the records, and the existence of a definable climatic niche for all the ticks examined in this study support the development of predictive models to estimate the range of ticks and their projected changes under future climate scenarios. The modeled probability of occurrence for ticks affecting humans is shown in Figure 6 for the year 2020. Projections into the HLZ for future climate scenarios (2040, 2060, 2080, and 2100) are included in Figure 7A–D. Consequent with the projections of climate suitability for ticks, three major patterns emerge: (1) increasing suitability for human-biting ticks at northern latitudes, particularly in Europe and western Russia, and to a lesser extent in the northern United States and southern Canada; (2) a progressive loss of suitable conditions in central and southern Africa; and (3) increasing suitability along the eastern coast of Australia.

**Figure 8:**
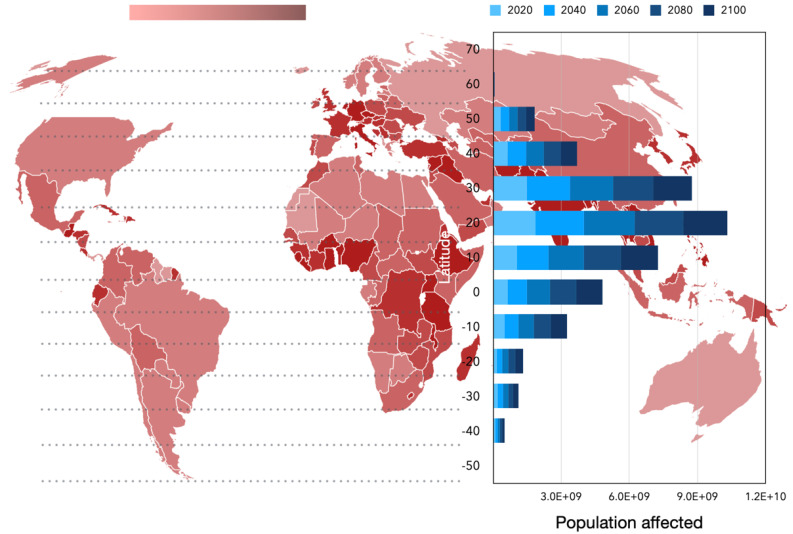
Predicted modeled impact of ticks and climate changes on humans in 2100. Countries are plotted in a range of red colors according to the net change in the expected climate suitability for ticks in the complete territory. The darker the color, the higher the expected net gain in suitability (gains—losses). The histograms on the right represent the number of persons at risk in the different slices of time (years 2020–2100) and are calculated as the population of the country weighted by the calculated climate suitability.

The trend of change in the climate suitability for these species of ticks is clear and summarized in Figure 8, in which the net rate of changes (gains minus losses) is displayed. The maps indicate that major net gains in suitability for tick human parasites are observed in central Europe and southern Canada, with smaller regions in sub-Saharan Africa, northern South America, and the Andes range; a large region in central Africa is expected to decrease in suitability for ticks in terms of climate.

We evaluated the impact on humans by estimating the number of people projected to live within tick-affected areas at each time period, stratified by latitude (Figure 8), and plotted as countries. The largest impact is expected between 30° and 40° N, encompassing the Mediterranean Basin, northern Mexico, the central United States, and parts of India and China. The increase in exposed population is proportional across all segments of the latitudinal profile. Although suitability increases markedly at high northern latitudes, the relatively low population densities in these regions result in a comparatively small impact. To note, the impact is weighted by the human population: the ranges with a high increase in suitability for ticks but scarce populations show a low impact.

The known distribution of ticks reported to affect livestock overlaps with major livestock production zones, including 54% of global cattle production, 49% of goats, and 51% of sheep, assuming the existing data for livestock density and the climate suitability of the 59 species of ticks included in the study. Areas of medium to high climatic suitability for ticks affecting livestock correspond to regions where densities exceed 17 cattle/km^2^, 16 sheep/km^2^, or 15 goats/km^2^. This means that more than 465 million cattle, 281 million sheep, and 408 million goats are being bred in areas with medium to high pressure from ticks.

**Figure 9:**
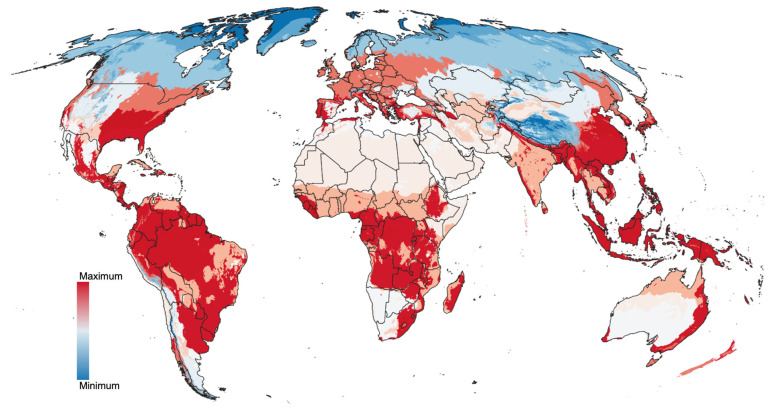
Modeled climate suitability for ticks affecting livestock in the baseline of the year 2020.

**Figure 10:**
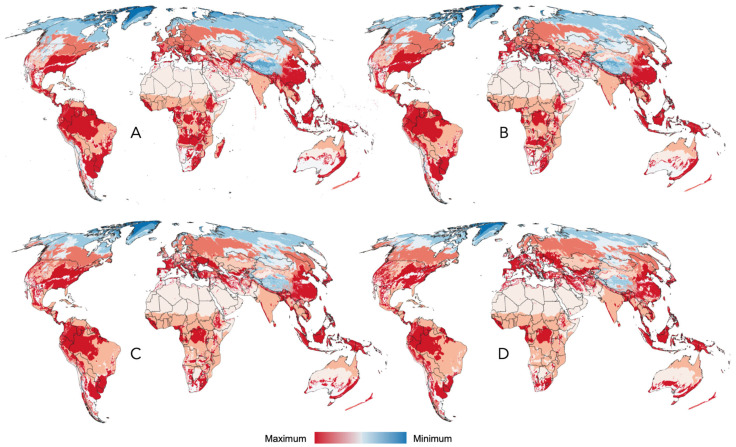
Modeled climate suitability for ticks affecting livestock. Results for the years 2040 (**A**), 2060 (**B**), 2080 (**C**), and 2100 (**D**).

The modeled climate suitability for ticks affecting livestock in the baseline of the year 2020 is shown in Figure 9. Projections of the climate suitability for tick parasites of livestock are included in Figure 10, reflecting a similar pattern to that observed for human-affecting species. The general rule is a sustained trend of increasing suitability in northen latitudes, with a lack of suitable zones in the Neotropics and the Afrotropical region. Ranges of livestock affecting ticks tend to decrease in wide regions of South America and southern Africa, with striking results for India without a noticeable change.

## 4. Discussion

### 4.1. Global Climatic Constraints on Tick Distributions

This study compiled the largest available dataset of tick distributions with reliable geographic coordinates and associated climate data, encompassing 138 species known to affect humans and/or livestock. By integrating these data into a global modeling framework, we assessed the potential impacts of climate change on human exposure to ticks. Our analyses were conducted at a broad spatial scale, explicitly addressing long-standing gaps in global knowledge of tick distributions. The primary objective was to quantify how climate-driven shifts in suitable habitat may translate into changes in human exposure risk through spatial redistribution of tick species. Parallel analyses were conducted for livestock, using official datasets of animal densities and explicitly distinguishing between ticks affecting humans and those affecting livestock.

It is important to emphasize the scope and limitations of this study. We focus on climate-driven changes in the potential geographic distributions of ticks, rather than on changes in local abundance, host–tick interaction rates, or pathogen transmission dynamics. Processes such as host redistribution, land-use change, landscape fragmentation, and socioeconomic conditions may amplify or attenuate the patterns identified here at regional and local scales. Nevertheless, by isolating the climatic signal, this work provides a necessary macroecological baseline. Such a baseline is essential for the development of more mechanistic models that explicitly integrate hosts, pathogens, and human behavior, and for informing long-term surveillance, risk assessment, and adaptation strategies under ongoing climate change. To our knowledge, no previous study has assembled or made openly available a global dataset of tick records of comparable size, taxonomic breadth, and spatial reliability.

Climate is the first-order driver of global tick distributions. Our results provide strong support for climate as a primary determinant of the large-scale distribution of ticks parasitizing humans and livestock. Across 138 species and 10 genera of Ixodidae, tick occurrences are consistently associated with distinct regions of climatic space defined by temperature and atmospheric water balance. Multivariate analyses (MANOVA, redundancy analysis, RDA, and principal components analysis, PCA) demonstrate that genera occupy statistically separable portions of the climatic niche, indicating that broad-scale climatic constraints operate above local ecological processes. This finding aligns with previous global and continental-scale studies suggesting that temperature accumulation and measures related to desiccation stress are fundamental limits to tick survival and development [3,62]. At this scale, climate acts as a filtering mechanism that delineates where ticks can persist, even if local abundance is modulated by hosts, vegetation structure, or land use.

### 4.2. Conceptual Scope: Climate Suitability Versus Ecological Complexity

Climate is not the only driver of the vertebrate hosts of ticks or reservoirs of tick-borne pathogens. We firmly adhere to the prevailing dogma that tick-borne pathogens depend on an intricate network of relationships between ticks and the vertebrate reservoirs [9,25,26,28]. This extreme is commonly ignored in studies aimed at overstating the importance of climate change on human health, performing modeling exercises that use only the ticks as explanatory variables. The modeling of the expected range of tick-transmitted pathogens by the only effect of climate ignores the distribution of the reservoirs, which are the best markers of the range of pathogens [63,64,65,66,67,68,69,70]. In the absence of modeling reservoir populations [71,72], we recommend a re-evaluation of the reports about tick-borne pathogens that have been modeled over an ecologically unreliable framework [73,74]. Consequently, the effects of climate trends on tick-borne pathogens are far from foreseeable if exclusively obtained from climate scenarios and would suggest a revision of the models projecting rates of tick-borne pathogens based solely on climate, without the ecological interactions among ticks and vertebrates.

Tick genera span a wide gradient of annual precipitation, from arid to highly humid environments, but are constrained to a narrower range of potential evapotranspiration ratios and biotemperature. This apparent discrepancy may reflect the fact that precipitation influences ticks indirectly, through vegetation cover and microclimatic buffering, rather than as a direct physiological driver. Laboratory experiments have consistently shown that tick survival and development depend on the balance between temperature and atmospheric moisture, typically expressed as relative humidity or saturation deficit [74,75,76,77]. Rainfall, as captured in global climate layers, is therefore likely a coarse proxy for processes that operate at finer spatial and temporal scales. Our findings support the view that accumulated heat and desiccation stress, rather than total precipitation, are the dominant climatic constraints shaping tick ranges on the planetary scale. Ticks that expend their complete life cycle in caves or animal shelters may be the exception to this rule because their environmental niche cannot be tracked by coordinates or the prevailing climate features.

### 4.3. Climate Change–Driven Redistribution of Tick Suitability and Exposure Risk

By projecting tick distributions within the framework of Holdridge life zones, this study assumes a degree of temporal stability in the relationship between tick genera and broad climatic categories. Importantly, this approach does not attempt to predict fine-scale presence or abundance but rather captures first-order shifts in climatic suitability as climate zones reorganize spatially under future scenarios. This assumption is supported by the clear clustering of genera within HLZ space and by the physiological conservatism observed in tick responses to temperature and humidity across life stages [78,79,80,81]. Similar niche-tracking assumptions underpin many species distribution modeling approaches applied to arthropod vectors. While evolutionary adaptation and plastic responses cannot be excluded over multi-decadal timescales [81,82], the use of HLZ offers a biologically interpretable and computationally tractable framework for global analyses.

A methodological limitation of this study is the implicit assumption of temporal stability in the relationship between climate and biome structure inherent in the Holdridge Life Zone framework. The HLZ system assumes that long-term climatic variables define relatively stable ecological envelopes and that future climatic conditions will translate into analogous life zones with comparable functional constraints. While this assumption is widely used in global change studies, it does not account for potential lags, nonlinear responses, or novel climate-vegetation assemblages that may emerge under rapid climate change. Consequently, our projections should be interpreted as indicators of shifts in climatic suitability rather than as deterministic forecasts of biome or tick distributions. Despite this limitation, the HLZ framework provides a coherent and transparent basis for comparing climate-driven changes across regions and taxa, which is essential for global-scale assessments spanning multiple decades.

Projections under future climate scenarios reveal a pronounced geographic asymmetry in changes to tick climatic suitability. Gains are concentrated at higher northern latitudes, particularly in Europe, southern Canada, and parts of western Russia, whereas substantial losses are projected across central and southern Africa. These contrasting patterns reflect the interaction between baseline climatic constraints and the direction of climate change. In cold regions, warming relaxes thermal limitations on tick development and seasonal activity, facilitating range expansion and longer questing periods [83,84]. In already warm regions, however, further increases in temperature and evapotranspiration may exceed physiological thresholds by increasing mortality and reducing habitat suitability. Such non-linear responses underscore why climate change does not uniformly increase tick risk worldwide but instead redistributes it across regions.

By integrating modeled climatic suitability with gridded human population data, we show that the greatest increase in human exposure is not necessarily located in areas with the largest relative gains in suitability for ticks. Instead, impact is maximized where moderate-to-high increases in suitability coincide with high population density, particularly between 30° and 40° N. This includes the Mediterranean Basin, parts of North America, and densely populated regions of Asia. Conversely, large suitability gains in sparsely populated northern regions translate into relatively small absolute impacts. These results reinforce the importance of coupling ecological projections with demographic data when assessing public health relevance. They also highlight that climate-driven changes in tick distributions may exacerbate exposure in regions already experiencing high burdens of tick-borne diseases.

### 4.4. Implications for Livestock and Tick Community Reorganization

The overlap between current tick suitability and global livestock production is substantial, encompassing roughly half of cattle, sheep, and goat populations worldwide, as reported elsewhere [85]. The spatial patterns of projected suitability changes for livestock-associated ticks broadly mirror those observed for human-biting species, suggesting shared climatic constraints. In many cases, because the same species is a reported parasite of both humans and livestock, including cattle, goats, or sheep [86]. However, unlike human populations, reliable global projections of livestock distribution under future socioeconomic and climatic scenarios are lacking [87]. Livestock density is shaped not only by climate but also by land use, market forces, cultural practices, and policy decisions [88]. Consequently, future impacts on livestock could not be quantified with the same level of confidence. This limitation emphasizes the need for integrated assessments that combine climate projections with scenarios of agricultural adaptation and land-use change.

What is observed in the time series of climate suitability for ticks is a disruption of the communities of ticks. The communities of ticks consist of those species that tend to cluster around a common climate niche, accommodating ecologically similar groups of taxa. These tend to co-occur with the communities of vertebrates that are exploited as hosts. It has been demonstrated in silico that the combinations of groups of ticks and hosts result in an ecological construct that defines the climate niche that supports the circulation of some tick-borne pathogens [28]. The results of our study predict a change in these clusters, and presumably the hosts, which could result in new arrangements of the ticks and vertebrate partners that circulate the pathogens. We did not address the probable impact of climate change on tick-borne pathogens.

### 4.5. Gaps, Caveats, and Future Research Needs

Despite the unprecedented size of the compiled dataset, pronounced geographic gaps remain, notably across Russia, India, and parts of Central Asia. These gaps likely reflect uneven surveillance efforts and limited availability of georeferenced records rather than true absence of ticks. Such biases are a pervasive challenge in global biodiversity and disease ecology studies [89,90]. While our modeling framework mitigates some effects of sampling bias by focusing on climatic niches rather than raw occurrence counts, uncertainties remain in poorly sampled regions. Notably, countries well known to host important tick pests of livestock (e.g., India) lack adequate coverage; this is projected in the results as a “lack of impact” of the ticks in that territory, which is unreliable. Improving global tick surveillance, standardizing data collection, and increasing the availability of open-access georeferenced records are critical steps for refining future projections.

This study has gaps that limit the knowledge obtained. For example, there are no estimates of livestock populations under scenarios of climate change and for the complete Earth’s surface. Furthermore, there are no estimates of the cost of the anti-tick treatments for many countries and regions, and the availability of data is restricted to some studies and field situations [90,91]. Without estimations of anti-tick treatments on a global basis, we produced an index that considers the modeled abundance of ticks and the FAO-derived livestock density, together with an estimation of the relative changes in that impact per hexagon. An explicit analysis of the impact on human populations based on health indicators could not be included because a wide panoply of data exists at national levels, not projected into a regular grid as explored in the current study. We aimed to approach a solid ecological indicator driven by climate; we foresee a potential application of the widely accepted health indicators over these estimations of range gain or decrease.

We advocate for improvements in surveillance systems and capacity, investment in sustainable vector control tools, and fostering public-private partnerships to develop innovative interventions. By incorporating high resolution human health-derived indicators, a comparison between regions, countries, or demographic groups is allowed, and an early warning exercise is promoted. It could be possible to flag areas needing attention, acting as early indicators of potential health crises. Future studies should explicitly address a cost-benefit analysis, which is very necessary. The implementation of monitoring activities for ticks and transmitted pathogens should be overlapped with an estimation of costs, both direct (e.g., sanitary) and indirect (e.g., impact on working hours, direct costs to health systems). Other factors associated with tick resistance to chemical acaricides and the implementation of vaccines for the control of tick populations and TBDs should be considered.

## 5. Conclusions

This study provides a comprehensive global assessment of how climate change may reshape the distribution of ticks parasitizing humans and livestock. By integrating an unprecedented compilation of georeferenced records with a bioclimatic framework grounded in Holdridge life zones, we demonstrated that tick occurrence patterns are strongly structured by large-scale gradients of temperature and atmospheric water balance, but not rainfall. Projections under future climate scenarios consistently indicate a poleward expansion of climatic suitability, particularly in the Northern Hemisphere, alongside substantial losses in parts of Africa and other warm regions. When combined with human population projections, these shifts translate into heterogeneous changes in exposure risk, with the greatest impacts concentrated in densely populated mid-latitude regions rather than at the extremes of range expansion. Although the study does not address host dynamics, tick abundance, or pathogen transmission, it establishes a robust macroecological baseline against which more detailed process-based models can be developed. These results highlight and guide the need for integrating climate-driven range dynamics into long-term strategies for the surveillance, development, and implementation of sustainable control strategies, including vaccines for the control of tick infestations and transmitted pathogens, public health planning, and livestock management in a rapidly changing world.

## Figures and Tables

**Table 1:** The ticks are detected as indicator species of the HLZ. We used an algorithm weighing the specificity and the persistence of a species in an HLZ. Included are the only species that had a significant association with a given HLZ (including in the column with that name). The “stat” is the value of the test, and the *p*-value indicates the significance.

Species	HLZ	Stat	*p*-Value
*Amblyomma americanum*	21	0.448753	0.001
*Amblyomma geoemydae*	37	0.418042	0.017
*Amblyomma helvolum*	37	0.368887	0.028
*Amblyomma javanense*	37	0.382064	0.027
*Amblyomma maculatum*	3	0.366640	0.018
*Amblyomma varanense*	37	0.367643	0.031
*Dermacentor nuttalli*	4	0.324301	0.027
*Dermacentor silvarum*	8	0.343636	0.011
*Dermacentor variabilis*	21	0.355470	0.006
*Haemaphysalis longicornis*	13	0.296391	0.022
*Hyalomma dromedarii*	32	0.339285	0.01
*Hyalomma impeltatum*	32	0.306262	0.013
*Ixodes granulatus*	37	0.352572	0.033
*Ixodes holocyclus*	37	0.389487	0.026
*Ixodes ricinus*	16	0.329045	0.004
*Ixodes scapularis*	21	0.411829	0.001
*Ixodes uriae*	2	0.331969	0.047
*Rhipicephalus microplus*	37	0.333025	0.004
*Rhipicephalus rossicus*	23	0.351262	0.039
*Rhipicephalus turanicus*	17	0.327619	0.016

## Data Availability

All the raw data produced or compiled in this study are available at FigShare at the link https://figshare.com/articles/dataset/Dataset_of_the_paper_b_Scientist_s_opinion_on_climate_change_and_ticks_Ixodidae_b_/31081453 (accesed on 1 February 2026) with doi 10.6084/m9.figshare.31081453 (this doi is available once the paper is published). All the data are provided under the Creative Common License 3, in which they can be used for research, but the origin must be credited. If the data are modified they must be shared in the same way. If new research generated by these data is not openly shared, it will inflict the license. Credit to GBIF downloads is as follows: DOI10.15468/dl.p23xq4, DOI10.15468/dl.6bu4cv.

## Supplementary Materials

All the data produced in this study are available at the link https://figshare.com/articles/dataset/Dataset_of_the_paper_b_Scientist_s_opinion_on_climate_change_and_ticks_Ixodidae_b_/31081453, accessed on 2 January 2026. The DOI of the dataset is https://doi.org/10.6084/m9.figshare.31081453.
